# Long term outcomes after left atrial appendage closure with the LARIAT device—Stroke risk reduction over five years follow-up

**DOI:** 10.1371/journal.pone.0208710

**Published:** 2018-12-19

**Authors:** Radoslaw Litwinowicz, Magdalena Bartus, Marian Burysz, Maciej Brzeziński, Piotr Suwalski, Boguslaw Kapelak, Venkat Vuddanda, Dhanunjaya Lakkireddy, Randall J. Lee, Rafal Trabka, Krzysztof Bartus

**Affiliations:** 1 Department of Cardiovascular Surgery and Transplantology, Jagiellonian University, John Paul II Hospital, Krakow, Poland; 2 Departament of Pharmacology, Jagiellonian University, Krakow, Poland; 3 Department of Cardiac Surgery, Regional Specialist Hospital, Grudziadz, Poland; 4 Department of Cardiac and Vacsular Surgery, Medical University of Gdansk, Gdansk, Poland; 5 Department of Cardiac Surgery, Central Clinical Hospital of Ministry of Interior, Warsaw, Poland; 6 Warren Alpert School of Medicine, Brown University, Harvard Medical School, Boston MA, United States of America; 7 Division of Cardiovascular Diseases, Cardiovascular Research Institute, University of Kansas, Kansas City, KS, United States of America; 8 Department of Medicine, Division of Cardiac Electrophysiology, University of California San Francisco, San Francisco, CA, United States of America; 9 Cardiovascular Research Institute, Institute for Regeneration Medicine, University of California San Francisco, San Francisco, CA, United States of America; 10 Departament of Rehabilitation, Jagiellonian University, Krakow, Poland; University of Bologna, ITALY

## Abstract

**Introduction:**

Left atrial appendage closure (LAAC) with LARIAT offers an alternative to oral anticoagulation (OAC) for patients with atrial fibrillation. The aim of this study was to present long-term clinical outcomes of LAAC in these patients (AF).

**Material and methods:**

A prospective, single-center study was performed in 139 patients undergoing LAAC with Lariat. Thromboembolic events, severe bleeding and mortality rate were recorded. The reduction in risk of thromboembolism and bleeding after LAAC was calculated.

**Results:**

The mean CHADS_2_-score was 1.8 ± 1.0, mean CHA_2_DS_2_-VASc score was 2.9 ± 1.6 and HAS-BLED score was 3.1 ± 1.1. After 428.4 patient-years of follow-up (mean 4.2±1.0 years), the thromboembolism rate was 0.6% with a calculated thromboembolism risk reduction of 81%. The severe bleeding rate was 0.8%; calculated bleeding risk reduction was 78%. The overall mortality rate was 1.6%.

**Conclusions:**

Long-term outcomes show that LAAC with Lariat is a safe and effective treatment for stroke prevention and bleeding risk reduction in AF patients with a high level of underlying risk.

## Introduction

Left atrial appendage closure (LAAC) for stroke prevention in patients with atrial fibrillation (AF) is one of the fastest developing branches of interventional cardiology with the number of procedures rising annually. Currently, LAAC procedures are a recommended alternative for AF patients in whom oral anticoagulation (OAC) therapy is ineffective or contraindicated [1,2].

Exclusion of the left atrial appendage (LAA) may be performed using endocardial (Watchman, Amplatzer) or epicardial (Lariat) devices [3]. In contrast to endocardial devices, epicardial devices is an non-implant solution with does not inhibit a post LAA closure ablation procedure.

Long-term outcomes of AF patients treated with endocardial devices have demonstrated the safety and efficacy of this approach [4,5]. However, long-term outcome studies for epicardial devices are currently lacking. [3,6].

In this study we evaluated the long-term outcomes of patients with AF treated with the Lariat device in a single center.

## Materials and methods

All patients were fully informed about the procedure and gave written informed consent. The protocol was conducted with the approval of the Polish Ministry of Health and the ethics committee at John Paul II Hospital, Krakow, Poland.

A prospective, single-center study was performed in 139 patients, who were referred between December 2009 and December 2012 for LAAC with the LARIAT device. Left atrial appendage occlusion with the first generation of LARIAT device (SentreHEART Inc, Redwood, CA) has been described in detail in previous studies [7–10]. Briefly, the procedure is performed with three main components: a compliant occlusion balloon, two magnet-tipped guide wires, and a 12-Fr suture delivery device. Following percutaneous pericardial approach, trans-septal puncture is performed. The first endocardial magnet-tipped guidewire is placed near the apex of the LAA. Using percutaneous femoral access, the second endocardial magnet-tipped guidewire is placed at the tip of the LAA to establish a stable connection between the wires. The LARIAT snare device is then advanced over the epicardial guidewire to occlude the LAA. After TEE and fluoroscopic confirmation of LAA closure a pre-tied suture is deployed and tightened to ligate the LAA. During procedure no periprocedural colchicine for pericarditis prophylaxis were used [3,7,8].

### Post-procedure anticoagulation

Antiplatelet therapy with aspirin was recommended for all patients. However, final therapy varied due to patient comorbidities, contraindications, and physician preference.

### Thromboembolism and bleeding reduction calculation

Adverse events reported during follow-up visit, based on the Munich consensus document [11], included mortality (cardiovascular, non-cardiovascular, procedural mortality, immediate procedural mortality), thromboembolic events (stroke, TIA, systemic embolism) and bleeding (life threating or disabling, major bleeding, minor bleeding).

As in our previous study[12], individual patients’ annual risk was recorded, and the average annual risk for the entire study population was calculated. The total number of thromboembolic events during overall follow-up periods were divided by the total patient years of follow-up and were multiplied by 100 to get the actual annual rate of thromboembolic events. Thromboembolism reduction was calculated as follows: (estimated %—actual % event rate)/estimated % event rate) [12,13]. Procedure efficacy to prevent thromboembolic events (stroke, TIA, systemic embolism, thrombus in the heart chamber) was calculated by comparing the actual event rate with the event rate predicted by the CHA_2_DS_2_-VASc scores [1,14,15]. Annual risk was recorded both for individual patients and the study population overall [12,13].

Bleeding reduction was assessed in a similar manner [12,13], comparing actual events with the number predicted by the HAS-BLED score [16].

### Statistical analysis

Continuous variables were analyzed for normal distribution using the Shapiro-Wilk test. Data are expressed as mean ± standard deviation or median (interquartile range; Q1-25th percentile and Q3-75th percentile), unless otherwise stated. If a non-parametric test was used, the obtained data were additionally presented as mean ± standard deviation for better comparison with other studies. To assess the differences between two continuous variables, a Student’s t-test, or Mann-Whitney U-test were used as appropriate. Categorical variables were expressed as counts and percentages. Baseline characteristics between groups were compared using the t test for continuous variables and the chi-square test for categorical variables. Kaplan-Meier analysis was performed to estimate survival over time. Statistical analysis was performed with STATISTICA 10.0 (StatSoft, Tulsa, OK, USA). A two-sided p-value <0.05 was considered statistically significant.

## Results

Patients baseline characteristics are summarized in Table 1. The mean CHADS_2_-score was 1.8 ± 1.0, mean CHA_2_DS_2_-VASc score 2.9 ± 1.6 and HAS-BLED score 3.1 ± 1.1. Prior to the procedure 85.6% patients were taking VKA, 12.2% an antiplatelet agent and 2.2% received no anticoagulation.

**Table 1:** Baseline patient characteristics.

Variable	LAAO (n = 139)
Age, years	
[Mean ± SD]	61.75 ± 9.9
[Median (Q_1_±Q_3_)]	62 (56 ± 68.75)
[Range]	21–81
Female	46.0%
CHADS_2_ score	
[Mean ± SD]	1.8 ± 1.0
[Median (Q_1_±Q_3_)]	2(1± 3)
CHA_2_DS_2_-VASc score	
[Mean ± SD]	2.9 ± 1.6
[Median (Q_1_±Q_3_)]	3(2± 4)
HAS-BLED score	
[Mean ± SD]	3.1 ± 1.1
[Median (Q_1_±Q_3_)]	3(2± 4)
Chronic Heart Failure	12.9%
Coronary Artery Disease	18.7%
Diabetes mellitus	20.1%
Previous TIA/stroke	26.6%
History of ablation	9.4%
Hypertension	93.5%
Chronic Obstructive Pulmonary Disease	6.5%
Pacemaker	17.3%
Pre-procedure medications	
Vitamin K Antagonist	85.6%
Antiplatelet agents	12.2%
None	2.2%
LAA dimension	
LAA width [mm]	28(22 ± 32)
LAA length [mm]	30(25 ± 37)
Number of lobe	
1 lobe	44.7%
2 lobes	41.7%
3 ≥ lobes	13.6%
Indication for LAAC	
Stroke/TIA while on OAC	28.8%
Failure/complication* while on OAC	10.8%
Contraindicated to OAC	12.9%
Labile INR	47.5%

*Failure of OAC: history of left atrial/left atrial appendage thrombus despite OAC; complication of OAC: history of bleeding complication with OAC

Procedure related adverse events was noted in 3 (2.1%) cases. Two patients had a right ventricle puncture requiring drainage and observation resolved without cardiac surgery intervention. One patient developed a pericardial effusion during transseptal puncture due to superficial epigastric artery laceration requiring cauterization.

Total follow-up was 428.4 patient-years. During the study period, the average annual thromboembolic event rate was 0.6%: there was one episode of stroke and 2 episodes of thrombus in the LA (which resolved with unfractionated heparin with no further complications). The annual rate of severe bleeding complications was 0.8%: three episodes of hemorrhagic stroke and one episode of gastrointestinal bleeding. Overall annual mortality rate was 1.63%.

Detailed data are present in Table 2.

**Table 2:** Outcomes at follow-up.

Variable	LAAO (n = 139)
LAA closure	
Complete or < 1-mm leak	96.4%
< 2 mm leak	2.9%
< 3 mm leak	0.7%
< 1 year lost to follow-up	8.6% (n = 12)
Average follow-up	
Days	
[Mean ± SD]	1510 ± 362
[Median (Q_1_±Q_3_)]	1625 [1292 ±1757]
Months	
[Mean ± SD]	50.3 ± 12.0
[Median (Q_1_±Q_3_)]	54(43± 59)
Death	**5.5% (n = 7)**
Cardiovascular	3.9% (n = 5)
Non-cardiovascular	0.8% (n = 1)
Reason unknown	0.8% (n = 1)
Thromboembolic events	**2.4% (n = 3)**
Ischemic stroke/TIA	0.8% (n = 1)
Thrombus in LA	1.6% (n = 2)
Severe bleeding	**3.1% (n = 4)**
Post procedure medications	
Vitamin K Antagonist	44.1% (n = 56)
Vitamin K Antagonist + Aspirin	0.8% (n = 1)
New Oral Anticoagulants	14.4% (n = 20)
Aspirin	25.2% (n = 32)
LMWH	0.8% (n = 1)
None	13.4% (n = 17)

**Fig 1:**
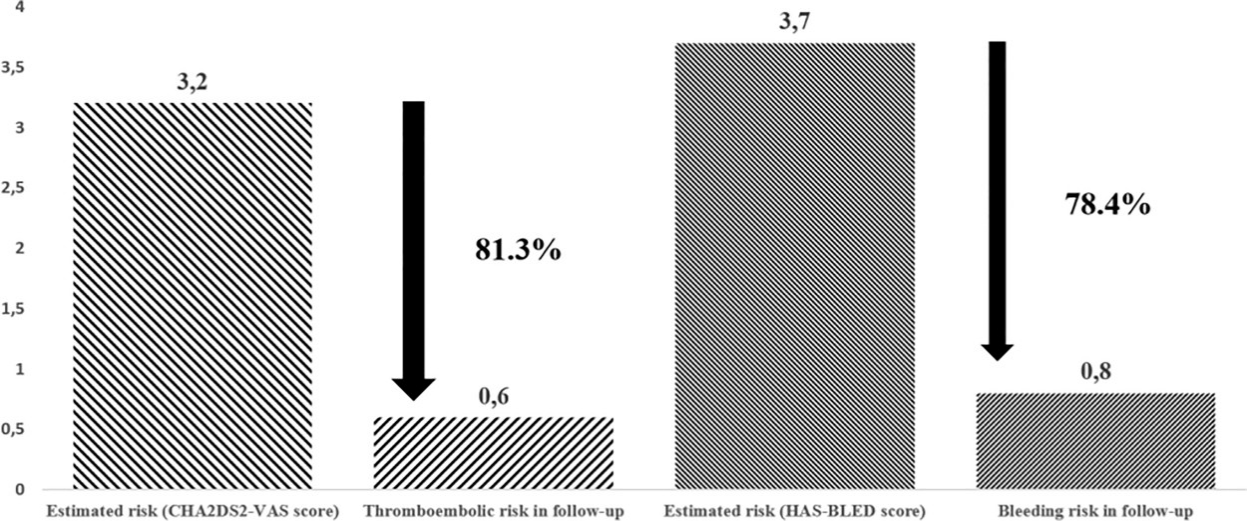
Effectiveness in stroke risk and bleeding reduction (/ 100 patient-years) during follow-up.

Estimated thromboembolic risk reduction and estimated bleeding risk reduction are presented in Fig 1.

## Discussion

This study demonstrates that the epidocardial approach for LAAC is safe and effective over long-term observations. We demonstrated significant reductions in the expected rate of stroke, thromboembolism and bleeding complications following LAAC for patients with AF.

Current ESC [1] and ACC/AHA/HRS [17] guidelines recommend OAC for stroke and thromboembolic prevention (class IA recommendation) in patients with AF and prior stroke or CHA_2_DS_2_-VASc scores ≤ 2. However, there is a large group of patients in whom OAC therapy is contraindicated or ineffective [1,17] or in whom OAC present fatal complications observed that are observed also in new oral anticoagulants[18]. In recent years LAAC has become an increasingly popular alternative [19–21]. ESC guidelines now include LAAC as a Class 2 B recommendation for patients with AF with a contraindication to OAC or a clinical history of severe bleeding [1]. Some cardiology associations are also starting to expand LAAC indications to include patients in whom OAC is ineffective [2].

The short-term safety and efficacy of the Lariat device has been demonstrated for diverse patient groups [7–10,12]. However, long term data regarding epicardial LAAC devices is limited.

In our patients the annual rate of stroke/TIA was 0.2% (one episode over 5 years). In similar 5-year follow-up studies the PREVAIL and PROTECT AF trials quoted a stroke rate of 1.7% for the Watchman device [22] and the PLAATO study reported 3.8% [5]. Although these numbers seem to favor the epicardial approach, we note that the mean CHADS_2_-score and CHA_2_DS_2_—VASc scores in this study were lower than in the endocardial study populations [5,22].

In this study we have demonstrated an 81.3% reduction in the expected rate of thromboembolic events. In the literature, there are only short-term observation study for stroke risk reduction after LAAC with endocardial devices. 1-year follow-up data of the EWOLUTION trail for Watchman device report 84% risk reduction for stroke prevention which is comparable with our results which present over 5 years observation [23]. In our opinion, due to the lack of randomized clinical trials in patients contraindicated for LAAO procedure, presented method of calculated risk reduction to the expected rate based on CHA_2_DS_2_-VASc score in the absence of stroke prevention therapy in this population seems to be a reasonable and comparable solution for evaluating the effectiveness of LAAC therapy.

We also calculated a 78.4% reduction in bleeding risk compared to HAS-BLEED predictions. In mentioned short-term results of EWOLUTION trail, the calculated bleeding risk reduction was 54% for Watchman device and was lower compared to our results[23]. However, it is difficult to accurately interpret this result. In contrast to an endocardial devices as the Watchman or the Amulet, epicardial exclusion with the Lariat uses a percutaneous approach to achieve suture closure of the LAA and leaves no foreign body inside left atrium [9,24]. In endocardial devices, the highest risk of thromboembolic events in the first 6 weeks post-procedure therefore VKA is recommended for 45 days after procedure, dual dual antiplatelet therapy for 6 months and aspirin thereafter [25,26]. After epicardial LAAC with Lariat permanent closure of the LAA is observed [24] therefore anticoagulation regimen is life-long aspirin treatment alone. Therefore our recommendation of aspirin monotherapy differs from the standard protocols following endocardial procedures [25,26]

It should be also mentioned, besides thromboembolic and bleeding risk reduction, epicardial LAAO present also hemodynamics and neurohormonal benefits [9,27]. In contrast to endocardial devices, after LAAO using epicardial access with the Lariat, there were significant increases in adiponectin and insulin, with decreased free fatty acids at 3 months post-procedure [27]. Additionally, N-terminal pro-A-type natriuretic peptide and N-terminal pro-B-type natriuretic peptide are significantly decreased in the acute phase after epicardial LAA device implantation, which subsequently normalized at 3 months [9,27]. Post endocardial LAA device implantation, the levels increased immediately and normalized after 24 h [27]. Additionally, with Lariat device there is a possibility to perform post LAAC ablation procedure.

Early experience with the Lariat procedure in some centers was associated with higher complication rates, related to epicardial access. It is important to notice that these findings were with early experience of the 1st generation Lariat procedure. The majority of procedures were performed with a large bore needle for pericardial access and no prophylactic use of colchicine. However, modification of the initial technique in Europe and also in the U.S.[6,28] to deploy a micropuncture needle and prophylactic colchicine has improved the safety profile of this device dramatically. The device itself has also been modified. The second generation of Lariat+ had larger snare accommodating LAA diameters up to 45 mm, steel braided shaft provides increased columnar strength within the shaft allowing it to overcome any influence of the epicardial sheath and distal marker of LARIAT for easy detection of correct orientation under fluoroscopy.

Our initial experiences with Lariat ^+^ devices showed 100% successful rate of complete LAA closure without any device or procedural related complications[29]. Comparable results of Lariat^+^ were also reported from other center with less experience with epicardial devices[30].

Analysis of the FDA MAUDE databases between May 2009 and May 2016 [31] has shown that LAAO was performed by the LARIAT approach in 4,889 cases. WATCHMAN was implanted in 2,027 patients prior to FDA approval in March 2015 and 3,822 patients post-approval. The composite outcome of stroke/TIA, pericardiocentesis, cardiac surgery, and death occurred more frequently with WATCHMAN (cumulative incidence, 1.93% vs. 1.15%; P = 0.001). Similar findings were observed for device embolization, cardiac surgery, and myocardial infarction. Our findings of an overall mortality rate of 1.63% confirm this as a safe procedure over long-term follow-up. However, analysis of the FDA MAUDE databases present some limitations. There was no comparison of patients profile, comorbidities, age, indications for LAA procedure, the risk of stroke or bleeding which may impact of obtained results.

## Conclusions

LAAC via an epicardial approach with the Lariat device is a safe and effective treatment in the management of AF patients; 5-year follow-up data shows a reduction in the calculated risk of stroke, thromboembolism and bleeding.

### Study limitations

This is a non-randomized, prospective, observational single center study with its inherent limitations. The major limitation for estimating the benefit of LAAC is the lack of a control group and relying on calculated stroke or bleeding risks for analysis. Only the first generation of Lariat devices were studied. Post-procedure anticoagulation was variable. This variability will have had an impact on thromboembolic and bleeding risk, and therefore our calculations of risk reduction.
